# A Novel Nickel-Foam/Tungsten-Powder/Epoxy-Resin Backing Material for Medical Ultrasound Transducers

**DOI:** 10.3390/s26092630

**Published:** 2026-04-24

**Authors:** Hao Wang, Yilei Li, Ke Zhu, Chenyang Zheng, Jinpeng Ma, Enwei Sun, Xudong Qi, Rui Zhang

**Affiliations:** 1 Functional Materials and Acousto-Optic Instruments Institute, School of Instrumentation Science and Engineering, Harbin Institute of Technology, Harbin 150080, China; 22b901035@stu.hit.edu.cn (H.W.); yilei_cyt@163.com (Y.L.); zhuke@mail.ioa.ac.cn (K.Z.); 21b901031@stu.hit.edu.cn (C.Z.); 20240251@hit.edu.cn (J.M.); sunew@hit.edu.cn (E.S.); 2 Key Laboratory of Science and Technology on Advanced Underwater Acoustic Signal Processing, Institute of Acoustics, Chinese Academy of Sciences, Beijing 100190, China; 3 Key Laboratory for Photonic and Electronic Bandgap Materials, Ministry of Education, School of Physics and Electronic Engineering, Harbin Normal University, Harbin 150025, China

**Keywords:** ultrasound transducer, nickel foam, acoustic attenuation, miniaturization

## Abstract

**Highlights:**

**What are the main findings?**

- A novel hybrid composite backing material was successfully developed by interpenetrating a three-dimensional open-cell nickel foam skeleton with a conventional tungsten-powder/epoxy-resin acoustic matrix.
- The optimized 100 PPI composite demonstrated exceptional acoustic attenuation (62.6 dB/cm at 5 MHz and 84.2 dB/cm at 7.5 MHz)—approximately tripling conventional capabilities—while maintaining an ideal acoustic impedance matching window of 10.81 MRayl.

**What are the implications of the main findings?**

- The integration of the continuous highly scattering metal skeleton facilitates a remarkable >60% reduction in the required axial dimension of the backing layer without degrading the transducer’s fractional bandwidth or pulse-echo response.
- This innovative structural strategy effectively overcomes the inherent physical contradiction between high acoustic impedance and high attenuation, providing a highly scalable material pathway for the extreme miniaturization of advanced medical ultrasound probes.

**Abstract:**

The miniaturization of medical ultrasound imaging transducers is currently limited by the thick backing layers required to dissipate backward acoustic energy. To address this, a novel hybrid composite backing material was developed by interpenetrating a three-dimensional open-cell nickel foam skeleton with a traditional tungsten-powder/epoxy-resin matrix. Two groups of composite samples with varying pores per inch (PPI) were fabricated, and their acoustic properties were systematically characterized. Experimental results indicated that the 100 PPI composite achieved macroscopic acoustic attenuation coefficients of 62.6 dB/cm at 5 MHz and 84.2 dB/cm at 7.5 MHz. These values are roughly three times higher than conventional backing materials, while maintaining a suitable acoustic impedance of 10.81 MRayl. A 5 MHz transducer utilizing a 5.0 mm layer of this proposed backing achieved a −60 dB two-way pulse-echo insertion loss, effectively eliminating backside interference with performance comparable to a 16.5 mm conventional backing. This structural strategy successfully reduces the required backing axial dimension by over 60% without compromising transducer bandwidth, offering a viable material solution for miniaturized ultrasonic transducers.

## 1. Introduction

The emergence of ultrasound imaging technology has significantly advanced the field of non-invasive biomedical diagnostics. This technology enables the three-dimensional visualization of internal anatomical structures, offering distinct advantages such as high resolution, real-time feedback, and safety due to the absence of radiation [1,2]. Piezoelectric-based ultrasonic transducers act as the core components of these systems, serving as the interface between the electronic transmit-receive circuitry and biological tissues. The primary function of ultrasonic transducers is to convert electrical energy into mechanical acoustic vibrations and vice versa [3,4]. Therefore, the performance characteristics of the transducers directly influence the quality of the resulting images. Recently, driven by the growing adoption of intravascular ultrasound (IVUS), intracardiac echocardiography (ICE), and endoscopic ultrasound (EUS), there has been a growing need to miniaturize transducer size. This trend is essential to accommodate ultrasound imaging techniques that access deeper and narrower cavities within the human body [5,6].

Typically, ultrasound imaging transducers are composed of a matching layer, an active piezoelectric element (often employing advanced or high-temperature stable piezoceramics to maximize electromechanical conversion), and a backing layer [7,8,9]. The matching layer is designed to reduce the acoustic impedance mismatch between the piezoelectric material and the target medium. The piezoelectric material functions as the active element for electromechanical conversion, while the backing layer provides high acoustic attenuation to dampen reflections from the rear surface. This backing structure primarily serves as a semi-infinite attenuating medium to maximize the absorption of acoustic energy radiated backward from the piezoelectric element [10,11]. If this backward energy is not dissipated effectively, it reflects into the piezoelectric layer, causing severe multiple interfacial reverberations within the transducer. These internal reverberations significantly lengthen the transmitted ultrasound pulse and reduce pulse purity. Consequently, this degradation substantially impairs the axial resolution and causes severe near-field blind zone interference. Currently, the physical bulk of the backing structure presents a major obstacle to transducer miniaturization, as the layer requires sufficient geometric length to effectively dissipate sound waves, while simultaneously possessing high acoustic impedance to facilitate impedance matching with piezoelectric materials.

To achieve effective suppression of backward acoustic waves, an ideal backing material must simultaneously satisfy two criteria: (i) High acoustic impedance. In engineering practice, the backing material typically requires an acoustic impedance in the range of 10 to 15 MRayl. This is to ensure sufficient transmission of acoustic energy while simultaneously maintaining adequate bonding strength, high attenuation characteristics, and machinability [12,13]. (ii) Extremely high acoustic attenuation. The acoustic energy transmitted into the backing must be completely dissipated to prevent residual waves from reflecting into the piezoelectric material [14,15]. However, there is a physical conflict between these two criteria. This inherent contradiction arises because materials with high acoustic impedance typically possess high density and tight intermolecular bonding, which minimizes energy loss; consequently, the restricted molecular motion and structural homogeneity inherent in these materials result in low acoustic attenuation. Therefore, to achieve the functional role of the backing, it is necessary to increase its geometric length to ensure the complete dissipation of sound energy.

Currently, the most widely used backing material in laboratory and industry is the traditional tungsten-powder/epoxy-resin two-phase composites. In these composites, the epoxy resin acts as the continuous matrix to provide necessary structural strength and adhesion, while the dispersed tungsten powder primarily increases the density and acoustic impedance of the composites [16,17]. However, the attenuation of high-frequency acoustic waves in traditional two-phase backing materials relies mainly on the viscoelastic hysteresis loss of the epoxy resin, along with weak geometric or Rayleigh scattering at the surfaces of the fine tungsten particles. This limited energy dissipation mechanism imposes a physical bottleneck on enhancing acoustic attenuation capabilities. According to industry consensus in ultrasonic transducer design and evaluation standards for pulse-echo noise reduction, the backing layer must provide a total two-way pulse-echo attenuation of at least −60 dB to eliminate imaging tail artifacts [18]. Owing to the low attenuation coefficient of the traditional tungsten-powder/epoxy-resin composites, achieving this stringent standard requires assembling with thick backing layers. For instance, a transducer with a center frequency of 5 MHz typically requires a backing layer thickness of 10.0 mm or even 15.0 mm. This severely restricts the miniaturization of medical imaging ultrasound transducers.

To overcome the thickness limitation and fundamentally improve attenuation, recent studies have incorporated low-density inclusions, such as hollow glass microspheres or polyamide resins, into the epoxy matrix [19,20]. This approach forms a three-phase composite containing a gas or low-density phase. The numerous micron-scale air voids significantly increase the acoustic scattering cross-section, thermal conduction, and viscous damping losses within the medium. Consequently, the acoustic attenuation coefficient in the megahertz range is greatly enhanced. However, the presence of these lightweight pores significantly reduces the overall density of the composite, causing a drastic drop in acoustic impedance (typically below 5 MRayl). This severe impedance mismatch between the backing materials and the piezoelectric layers leads to strong reflections of backward waves at the interface [21]; as a result, acoustic energy is trapped within the piezoelectric layer. This phenomenon induces pronounced tail artifacts, substantially reducing both the transducer bandwidth and the axial resolution.

In recent years, porous metals, particularly open-cell foams such as nickel foam characterized by three-dimensional interconnected topologies, have exhibited exceptional broadband sound absorption, noise isolation, and structural damping capabilities in the fields of architectural acoustics, noise mitigation, and industrial vibration control [22,23]. These properties are attributed to their high specific surface area, intricate pore networks, and excellent mechanical stiffness. Research indicates that the complex interconnected pore network within metal foams significantly increases the tortuosity of acoustic propagation. This forces elastic waves to traverse extended microscopic paths within a limited macroscopic thickness, thereby significantly amplifying the rates of viscous and thermal energy dissipation [24,25]. While the application of porous metals in low-impedance fluid (e.g., air) acoustics is well-established, their exploration within the field of medical ultrasound acoustics remains scarce. Nevertheless, the unique geometry and acoustic characteristics of metal foams suggest significant potential for their utility as backing materials in medical imaging transducers.

To exploit the inherent advantages of porous metals in acoustic attenuation, this study proposes a novel composite backing structure featuring a nickel-foam skeleton interpenetrated with a tungsten-epoxy matrix. The design incorporates a nickel foam skeleton integrated with a filler composed of epoxy resin and tungsten powder. Experimental results, evaluated at both 5 MHz and 7.5 MHz, indicate that the incorporation of the nickel foam skeleton significantly enhances sound attenuation characteristics. The underlying mechanism responsible for this improvement was investigated. Furthermore, to evaluate practical performance, 5 MHz ultrasound transducers utilizing the proposed hybrid composite backing and traditional tungsten-epoxy backing were fabricated and characterized to evaluate practical performance. The results demonstrate that this new hybrid structure allows for a substantial reduction in backing thickness while maintaining transducer performance comparable to, or even exceeding, that of conventional designs. These findings suggest that the proposed structural strategy offers a viable new paradigm for the development of high-attenuation acoustic materials and miniaturized transducer backing structures.

## 2. Materials and Methods

### 2.1. Preparation Process of Composite Samples

To evaluate the influence of the nickel-foam skeleton on the acoustic characteristics of the hybrid composites, two groups of samples were designed and fabricated for a comparative study.

Group-A (Nickel-foam/epoxy-resin two-phase composites):

These samples were fabricated to eliminate interference from heavy metal powders and independently assess the intrinsic acoustic dissipation of the nickel-foam skeleton within a pure polymer matrix. Initially, a nickel-foam skeleton of specific dimensions was secured in a customized mold. Subsequently, liquid epoxy resin, prepared at a standard ratio, was poured into the cavity. To mitigate bubbles introduced during pouring, the hybrid composite within the mold was placed in a high-vacuum degassing chamber for multiple cycles. Finally, the specimens were left static for 12 h at room temperature.

Group-B (Nickel-foam/tungsten-powder/epoxy-resin three-phase composites):

These samples were designed to satisfy the high acoustic impedance matching requirements of medical transducers. Initially, a tungsten-powder/epoxy-resin slurry with a tungsten powder weight percentage of approximately 80% was prepared and injected into the mold. Subsequently, the filled mold was secured within a custom high-speed centrifuge rotor, and then centrifugation was performed. The centrifugal force generated during rotation drove the high-viscosity slurry to penetrate and densely pack the three-dimensional skeleton of the nickel foam. This centrifugation process not only facilitated the deep infiltration of the micro-fillers but also effectively utilized the density difference to expel residual entrapped micro-bubbles from the matrix. Finally, the specimens were left static at room temperature.

Following the curing process, excess resin or unsupported tungsten powder at the mold periphery was removed using precision metallographic cutting equipment. Subsequently, the acoustic coupling surfaces of the samples were subjected to precision parallel grinding and then polished. For all specimens designated for acoustic testing, the parallelism of the end faces was strictly maintained within ±0.02 mm. This rigorous control eliminates non-intrinsic acoustic refraction errors resulting from surface inclination.

After the cutting and polishing processes, the specimens were standardized to a cross-sectional area of 28 × 28 mm and a thickness of 5.0 mm. In the context of ultrasonic testing at a center frequency of 5 MHz, this thickness corresponds to 9 to 14 times the acoustic wavelength in the composite medium. This geometry ensures strict bulk wave propagation, thereby mitigating thin-plate resonance effects.

### 2.2. Geometric Parameters of Nickel Foam

Nickel foams with distinct pore characteristics were systematically selected for this study. Pores per inch (PPI) characterizes the pore size and spatial network density of porous materials. A higher PPI value reflects more pores per unit length and implies a smaller average pore diameter. As a result, the metal ligaments making up the three-dimensional framework are finer and more closely packed [26]. Relative density (*ρ*_r_) is defined as the ratio of the nickel-foam density to the theoretical density of its constituent matrix (solid pure nickel), expressed as a percentage. A higher *ρ*_r_ signifies a denser metallic skeleton, leading to a significant increase in the total area of solid–solid acoustic impedance mismatch interfaces. These interfaces are formed between different media, such as epoxy and nickel, or tungsten-powder-filled epoxy and nickel. The physical properties and specifications of the raw materials used to fabricate the composite backing are summarized in Table 1.

### 2.3. Measurement System and Theoretical Calculation Methods of Acoustic Parameters

**Figure 1:**
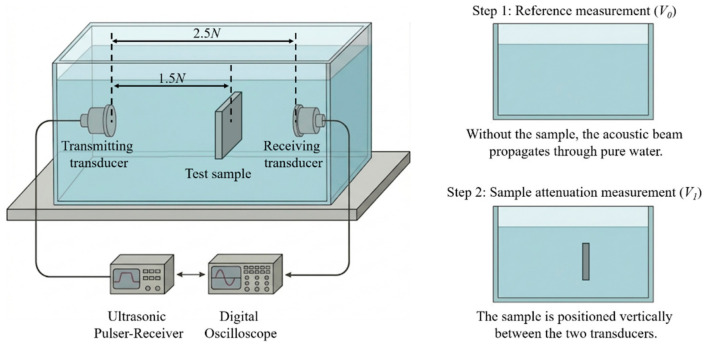
Schematic diagram of the measurement system for the acoustic attenuation coefficient.

Thin composite samples, each with a thickness of 2.0 mm, were fabricated to measure the sound velocities (c) using the pulse-echo method. Sample density (*ρ*) was determined through the Archimedes method. Subsequently, the acoustic impedance (*Z*) was calculated using the equation *Z* = *ρ* × *c*. The ultrasonic attenuation coefficient (*α*) was measured using a through-transmission water immersion method, as illustrated in Figure 1. At room temperature, two focused ultrasonic transducers (functioning in transmitter and receiver mode, respectively) were aligned coaxially on opposite sides of the sample. To ensure sufficient time-domain isolation and minimize phase cancellation errors, the receiver was positioned at a distance of 2.5 *N* from the transmitter, where *N* represents the near-field distance [27]. The measurement process is divided into two steps. First, a baseline reference peak voltage (*V*_0_) was recorded without the sample to establish the initial amplitude. The samples were then inserted vertically into the beam path, with their center positioned at 1.5 *N* from the transmitter. The resulting transmitted peak voltage (*V*_1_) was then recorded.

In this study, the acoustic attenuation coefficients of the samples were measured at 5 MHz and 7.5 MHz, respectively. The transmitting and receiving transducers employed featured an effective sound-emitting diameter (*D*) of 13 mm. For such an unfocused, disk-shaped transducer, the near-field distance (*N*) demarcating the boundary between the near and far fields was calculated using the classical diffraction equation [28]:

(1) $$N=\frac{D^{2}}{4\lambda }=\frac{D^{2}\times f}{4c_{w}}$$

In this equation, *N* represents the near-field distance of the ultrasonic beam, and *D* is the effective diameter of the piezoelectric element inside the transducer. The *λ* denotes the acoustic wavelength in deionized water, while *f* is the center frequency of the ultrasound source. The *c*_w_ refers to the longitudinal sound velocity in deionized water at room temperature, which is taken as 1480 m/s. Based on Equation (1) and the given conditions of deionized water immersion, the near-field distances of the transducers in this system are calculated to be *N*_5MHz_ = 142.7 mm and *N*_7.5MHz_ = 214.1 mm.

By recording the transmitted voltage *V*_0_ and *V*_1_, the acoustic attenuation coefficient α of the composite materials is calculated using the following equation [29]:

(2) $$\alpha =\frac{200}{d}\times \log_{10}(\frac{V_{0}}{V_{1}}\times \frac{4Z_{w}Z_{s}}{(Z_{w}+Z_{s}{)}^{2}})$$

where *α* is the acoustic attenuation coefficient (dB/cm), and *d* is the sample thickness (mm). *V*_0_ denotes the peak voltage measured by the receiver without the sample, while *V*_1_ refers to the transmitted peak voltage after attenuation by the measured sample (mV). In this study, the *V*_0_ measured at 5 MHz and 7.5 MHz were 501 mV and 503 mV, respectively. The terms *Z*_w_ and *Z*_s_ correspond to the acoustic impedances of deionized water and the sample, respectively (MRayl). *Z*_w_ is taken as 1.5 MRayl.

Given that the samples are highly heterogeneous hybrid three-phase composites, acoustic wave propagation within them is primarily governed by multiple scattering and interfacial mode conversion. Consequently, microscopic single-phase intrinsic attenuation mechanisms cannot accurately describe the overall acoustic behavior of the materials. Therefore, the density and acoustic attenuation parameters determined in this study refer specifically to the “macroscopic apparent density” and “macroscopic effective acoustic attenuation coefficient”. In this analysis, the entire 5.0 mm thick composite is treated as an equivalent macroscopic continuous medium. The focus is placed on evaluating the global acoustic wave dissipation and transmission performance, which are dominated by complex spatial structure and multiphase interfaces of hybrid composites [30].

### 2.4. Transducer Fabrication and Performance Characterization

The fabrication process for the 5 MHz medical ultrasound transducers in this study is detailed as follows:

(1) Selection of Piezoelectric Elements: Three high-consistency PZT-5H piezoelectric ceramics were selected as piezoelectric layers. This ensured experimental reproducibility. Each sample had a 12.000 mm diameter and a 1.520 mm initial thickness. The measured sound velocity was 4438 m/s, and the density was 7.614 g/cm^3^. The acoustic impedance was 33.791 MRayl. The piezoelectric constant *d*_33_ was 687 pC/N. Resonance and anti-resonance frequencies were 1.295 MHz and 1.475 MHz, respectively. All key performance deviations were within ±2%.

(2) Damping Ring Fabrication and Electrode Fixation: The piezoelectric ceramic and the H65 copper electrode pillar were bonded to a metallographic grinding stage. Subsequently, a damping ring was fabricated by casting an Al_2_O_3_/epoxy resin composite. This composite featured an Al_2_O_3_ mass ratio of 20%. The damping ring served three primary functions. First, it provided lateral physical constraints to suppress radial vibrations. This mechanism optimized the spectral purity of the pulse-echo signals. Second, it secured the ceramic to the electrode pillar. This fixation ensured the mechanical stability of the electrical connection. Finally, it provided structural support for the brittle piezoelectric plates during subsequent high-intensity precision grinding.

(3) Fabrication of 1–3 Piezoelectric Composite Layer: Following the full cure of the damping ring, a precision dicing saw micro-structured the piezoelectric ceramic into a 1–3 composite configuration. A diamond blade with a thickness of 0.050 mm was employed to produce kerfs with a pitch of 0.300 mm. The dicing process included two orthogonal cutting sequences. Subsequently, the resulting kerfs were backfilled with the same Al_2_O_3_/epoxy resin composite. A vacuum degassing procedure was performed during the filling process. This step ensured the complete penetration of the polymer and eliminated micro-bubbles. Finally, a 1–3 composite layer with a periodic ceramic pillar array was successfully realized.

(4) Electrode Deposition and Matching Layer Preparation: The epoxy resin in the 1–3 composite reached full cure. Subsequently, the piezoelectric layer was thinned to approximately 0.600 mm via precision grinding. This step ensured high surface planarity. A 0.010 mm thick gold (Au) layer was then deposited via magnetron sputtering. This gold layer established electrical connectivity between the ceramic surface and the copper electrode pillar. Subsequently, a matching layer with a thickness of 2.000 mm was cast using the 20% Al_2_O_3_/epoxy mixture. The measured acoustic impedance of this layer reached 3.633 MRayl. This value closely aligns with the theoretical optimum (3.672 MRayl) from the KLM model. Multiple vacuum degassing cycles were conducted to eliminate micro-bubbles. This direct-cast process ensured a tight physical bond between the matching and piezoelectric layers. Moreover, it eliminated the requirement for an additional bonding layer.

(5) Precision Grinding and Backing Integration: The matching layer was thinned to 0.139 mm via precision parallel grinding. This thickness corresponds to the quarter-wavelength at 5 MHz. Subsequently, the sample was reversed and bonded to the metallographic grinding stage. The thinning process of the piezoelectric layer continued. Finally, this layer reached the theoretical design value of 0.360 mm. Next, the copper electrode pillar was shielded. A second 0.010 mm thick gold (Au) layer was then sputtered. Subsequently, the pre-fabricated backing sample was integrated onto the piezoelectric layer. Vacuum-degassed pure epoxy resin served as the bonding layer. A constant weight of approximately 5 kg was applied during assembly. This pressure strictly controlled the thickness and parallelism of the bonding layer. This uniform compression reduced the bonding layer to an ultra-thin state of approximately 0.015 mm. This thin layer minimizes acoustic interference and ensures efficient energy transmission into the backing. The backing layer fully covered the piezoelectric layer. Meanwhile, it maintained no contact with the electrode pillar. This design prevents electrical short-circuiting.

The performance of the fabricated transducers was characterized using the underwater echo method. The experimental setup included an ultrasonic pulse generator/receiver (Olympus 5073PR, Olympus, Waltham, MA, USA) and a digital oscilloscope (Tektronix TDS460A, Tektronix, Beaverton, OR, USA). Measurements were conducted in distilled water at room temperature, utilizing a polished steel block as the reflection target. The center frequency and −6 dB fractional bandwidth were determined by performing a Fast Fourier Transform (FFT) on the gated time-domain pulse-echo signals. Detailed characterization protocols and system calibration procedures follow the methodologies described in our previous work [31].

## 3. Results and Discussion

### 3.1. Acoustic Property Analysis of Nickel-Foam/Epoxy-Resin (Group-A) Samples

**Figure 2:**
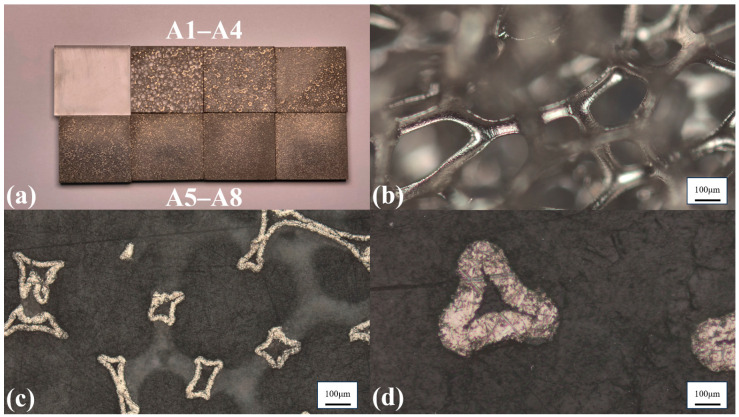
(**a**) Samples A1 to A8; (**b**) Epoxy-free 130-ppi nickel foam framework; (**c**) 10× magnified surface of sample A8 (130 ppi); (**d**) 50× magnified surface of sample A8.

Group-A binary hybrid composite samples (nickel-foam/epoxy-resin) are designed to investigate the influence mechanism of the nickel-foam skeleton on the acoustic properties of the hybrid composites. Figure 2 displays the physical object image and structural schematics of the prepared Group-A samples.

**Figure 3:**
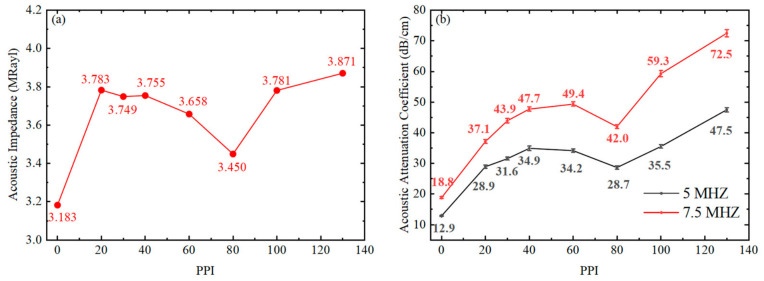
Acoustic properties of Group A samples versus PPI: (**a**) Acoustic impedance and (**b**) acoustic attenuation coefficient. The error bars in (**b**) represent the standard deviation.

It is observed that the samples exhibit a dense, pore-free matrix structure, with the epoxy resin fully infiltrating the nickel skeleton. Additionally, the exposed nickel skeleton on the surface is clearly visible under optical microscopy. These phenomena confirm the feasibility of the fabrication process for the nickel-foam/epoxy-resin composites. The pore size, density, and acoustic velocity were characterized, as summarized in Table 2. Figure 3a,b show the variation in acoustic impedance (*Z*) and acoustic attenuation coefficient (*α*) as a function of the PPI. The error bars in Figure 3b represent the standard deviation of the acoustic attenuation coefficient. In this case, the PPI value is inversely proportional to the pore size of nickel foam, which plays a critical role in determining the acoustic scattering and attenuation characteristics of hybrid composites.

As shown in Table 2, the density of the fabricated binary composites exhibits obvious variation with the increasing pores per inch (PPI) of the nickel foam. In general, the density shows an upward trend because the high-density nickel skeleton (8.908 g/cm^3^) replaces an equivalent spatial volume of the low-density epoxy resin (1.186 g/cm^3^). However, the density of the hybrid composite is also influenced by the relative density of the nickel skeleton. For instance, the nickel skeleton in the A6-sample exhibits a notably low relative density of 1.73%. This results in a reduced solid volume fraction of the heavy metal skeleton, causing the density of the A6-sample to deviate from the general trend and drop to 1.317 g/cm^3^. Meanwhile, as the specific mass fraction of the rigid metal skeleton increases, the acoustic velocity of the hybrid composite decreases from 2683 m/s (A1-sample) to 2535 m/s (A8-sample). Consequently, due to the competing effects of density and sound velocity, the acoustic impedance exhibits an irregular trend, as shown in Figure 3a. Nevertheless, compared to pure epoxy resin without a nickel skeleton, the acoustic impedance of the hybrid composites shows significant improvement.

As shown in Figure 3b, the acoustic attenuation coefficient exhibits an overall increasing trend with higher PPI, demonstrating a consistent positive correlation at both 5 MHz and 7.5 MHz. As a homogeneous material, the pure epoxy resin sample (A1) derives its attenuation solely from viscous absorption within the polymer, resulting in extremely low coefficients (12.9 dB/cm at 5 MHz and 18.8 dB/cm at 7.5 MHz). In contrast, the introduction of the nickel foam significantly enhances acoustic attenuation. This overall increasing trend results from the combined effects of solid–solid acoustic impedance mismatch, skeleton scattering, and spatial interference of characteristic dimensions.

Primarily, the dominant mechanism is the massive number of solid–solid acoustic impedance mismatch interfaces introduced by the nickel foam. A nearly 15-fold impedance step exists between metallic nickel (approximately 50 MRayl) and the epoxy resin matrix (approximately 3.2 MRayl). According to acoustic interfacial reflection theory, when plane acoustic waves propagate to the interface of media with distinct acoustic impedances, the severe impedance mismatch prevents smooth transmission, forcing intense interfacial transmission, reflection, and mode conversion. The proportion of reflected energy is directly proportional to the degree of impedance mismatch. This multiple scattering mechanism, dominated by extreme impedance mismatch, effectively confines acoustic waves within the local network for repeated attenuation, thereby greatly increasing the overall acoustic attenuation coefficient of the samples [32].

Secondly, the metallic struts of the nickel foam act as continuous rigid cylinders, forming strong scattering centers, while the epoxy resin within the pores constitutes a continuous phase matching the wavelength. The macroscopic average sound velocity of the Group-A samples is approximately 2640 m/s, which closely aligns with the sound velocity of pure epoxy resin (Sample A1) and differs significantly from that of pure nickel (approximately 5600 m/s). This indicates that acoustic waves propagate primarily along the continuous epoxy resin medium, with the internal nickel foam skeleton serving as strong scatterers in the propagation path. For Group-A samples, the wavelengths of 5 MHz and 7.5 MHz ultrasound are approximately 528 μm and 352 μm, respectively.

As the PPI changes, the relative relationship between the pore size of the divided continuous phase and the acoustic wavelength, along with the variation in strut diameter, jointly determine the nonlinear evolution of the acoustic attenuation coefficient across different PPI ranges:

In the 20 to 40 PPI range (Samples A2 to A4), the average pore size of the samples (1270 to 635 μm) is significantly larger than the testing wavelengths, placing the system in the independent scattering regime. Here, the increase in the number of solid–solid impedance mismatch interfaces per unit volume, brought by the higher PPI, plays an absolutely dominant role. Consequently, the acoustic attenuation coefficient exhibits a monotonic increase with the maximum slope as the number of interfaces grows.

In the 40 to 60 PPI range (Samples A4 to A5), the average pore size decreases to 423 μm. For 5 MHz (wavelength 528 μm), the continuous phase pore size crosses the wavelength dimension into the sub-wavelength regime. Destructive interference occurs among scattered waves from adjacent interfaces. Energy that would otherwise dissipate outward is forced back into the main acoustic beam, leading to a saturation of total scattering efficiency [33]. As a result, the attenuation curve for 5 MHz exhibits a stagnation plateau here (dropping from 34.9 to 34.2 dB/cm). Conversely, for 7.5 MHz (wavelength 352 μm), the pore size remains larger than the wavelength and has not fully entered the coherent cancellation zone; thus, the attenuation coefficient continues to rise, forming a distinct frequency divergence phenomenon.

When the PPI reaches 80 (Sample A6), both curves display a significant abnormal drop (plummeting to 28.7 dB/cm and 42.0 dB/cm, respectively). At this point, the average pore size (318 μm) fully enters the sub-wavelength regime for both frequencies, and the strut diameter decreases to a very fine 40 μm, causing a precipitous decline in the scattering cross-section provided by the nickel foam skeleton. Crucially, this sample possesses an exceptionally low relative density of 1.73%, indicating a scarcity of physical skeleton acting as strong scattering centers. The combination of extremely weak individual scattering capability and sparse skeleton structures leads to a sharp regression in macroscopic acoustic attenuation.

In the 100 to 130 PPI range (Samples A7 to A8), although the pore size (195 μm) further decreases, the extremely high PPI provides a massive number of solid–solid acoustic impedance mismatch interfaces. The intensely dense physical division of interfaces forcibly compensates for the disadvantage of weakened individual scattering capacity at sub-wavelength dimensions. The surge in multiple reflections pushes the acoustic attenuation to the peak of the entire group, ultimately reaching 47.5 dB/cm (5 MHz) and 72.5 dB/cm (7.5 MHz) at 130 PPI.

In summary, introducing a nickel foam skeleton into the epoxy resin matrix effectively enhances the acoustic attenuation coefficient of the material. Strong impedance mismatch causes the coefficient to generally increase with higher PPI; however, the destructive interference triggered by pore sizes entering the sub-wavelength regime, combined with weakened individual scattering due to finer strut diameters, jointly account for its non-monotonic fluctuation pattern.

### 3.2. Acoustic Property Analysis of Nickel-Foam/Tungsten-Powder/Epoxy-Resin (Group-B) Samples

**Figure 4:**
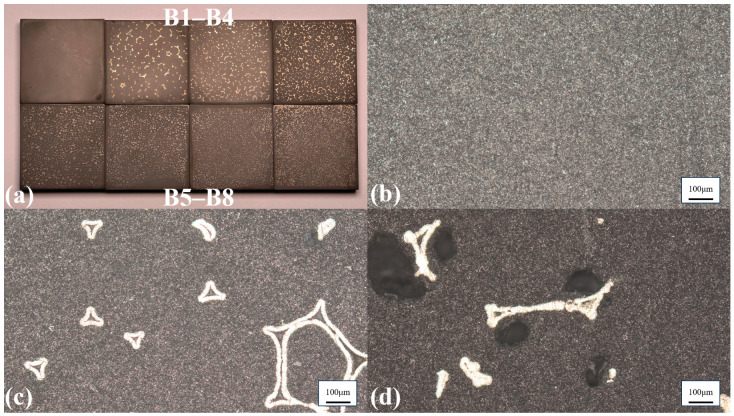
(**a**) Samples B1 to B8; (**b**) 10× magnified surface of sample B1 (conventional backing); (**c**) 10× magnified surface of sample B7 (100 ppi); (**d**) 10× magnified surface of sample B8 (130 ppi).

Based on the verification that the nickel foam skeleton can effectively improve the sound attenuation characteristics; this work further introduces a tungsten powder dispersed phase into the epoxy resin matrix to fabricate Group-B three-phase hybrid composites. This design is intended to enhance the acoustic attenuation and acoustic impedance of the designed backing structure for practical application in ultrasound imaging transducers. Improved sound attenuation facilitates the miniaturization of the backing structure. Meanwhile, the increased acoustic impedance is used to achieve acoustic impedance matching between the piezoelectric materials and the backing layer. The weight percentage of tungsten powder (3–5 μm particle diameter) is approximately 80% in the fabricated nickel-foam/tungsten-powder/epoxy-resin hybrid composites. Figure 4a displays the fabricated Group-B samples, while Figure 4b–d present the B1, B7, and B8 samples at a 10× magnification, respectively. It can be observed that the B1 and B7 samples similarly exhibit a dense and pore-free matrix structure, with the mixture of epoxy resin and tungsten powder fully infiltrating the nickel skeleton in the B7 sample. In contrast, large areas containing exclusively epoxy resin appear on the surface of the B8 sample.

**Figure 5:**
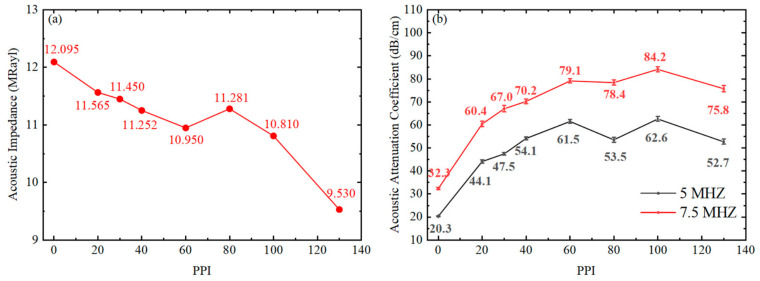
Acoustic properties of Group B samples versus PPI: (**a**) Acoustic impedance and (**b**) acoustic attenuation coefficient. The error bars in (**b**) represent the standard deviation.

Table 3 summarizes the pore size, density, and acoustic velocity of the Group-B hybrid composites. It indicates that the traditional backing material without nickel foam (B1-sample) exhibits a high density of 6.868 g/cm^3^. The introduction of nickel foam results in an overall downward trend in density, influenced by two primary factors. First, the density of the nickel skeleton (8.908 g/cm^3^) is significantly lower than that of the tungsten powder (19.254 g/cm^3^). Second, as PPI and relative density increase, the pore size of the skeleton decreases. Although the nominal particle diameter of the tungsten powder is 3–5 μm, the particles are prone to agglomeration, resulting in a larger effective physical size. These enlarged agglomerates are blocked by the dense nickel skeleton. Consequently, a greater volume within the skeleton is occupied by the lower-density epoxy resin. Additionally, density variations are affected by the relative density of nickel foam. For instance, the B6-sample exhibits an abnormally low relative density of 1.73%. This implies that the skeleton occupies a smaller volume, permitting more high-density tungsten powder to substitute, which leads to a slight rebound in density to 6.468 g/cm^3^. However, as PPI continues to increase, this blocking effect becomes pronounced in the B8-sample (130 PPI). The extremely small pores prevent a significant amount of tungsten powder from entering, causing a sharp drop in the density of the B8-sample to 5.400 g/cm^3^. Meanwhile, since the acoustic velocities of the Group-B samples remain approximately 1750 m/s, their acoustic impedances stay within the optimal matching window of 10–15 MRayl, except for the B8 sample, which shows a substantial decrease, as shown in Figure 5a.

Figure 5b shows the variation in the acoustic attenuation coefficient as a function of the PPI value for Group-B samples measured at 5 MHz and 7.5 MHz. It can be observed that the trend in the Group-B hybrid composites is generally consistent with that of the Group-A samples, remaining fundamentally controlled by the PPI and the corresponding pore size. Using the B1-sample, representing the traditional backing material, as a baseline, it exhibits moderate attenuation coefficients of 20.3 dB/cm (5 MHz) and 32.3 dB/cm (7.5 MHz), relying primarily on weak Rayleigh scattering induced by dispersed tungsten particles. With the incorporation of the nickel foam skeleton and the increase in PPI, the decreasing pore size significantly increases the number of acoustic impedance mismatch interfaces (epoxy-tungsten/epoxy-nickel/tungsten-nickel). Driven by this mechanism, the acoustic attenuation coefficient of the Group-B samples steadily rises, peaking at 62.6 dB/cm (5 MHz) and 84.2 dB/cm (7.5 MHz) for the 100 PPI (B7-sample).

However, owing to severe blockage of the tungsten powder, the B8-sample experiences a reduction in high-density tungsten powder content, resulting in a decrease in the attenuation coefficient to 52.7 dB/cm. When comparing the results at 5 MHz and 7.5 MHz, the trends are highly consistent. Due to the shorter acoustic wavelength, the attenuation capability at 7.5 MHz is consistently stronger. In summary, for nickel-foam/tungsten-powder/epoxy-resin hybrid composites, moderately increasing the PPI reduces the pore size and improves acoustic attenuation. However, excessively high PPI leads to the blockage of heavy metal powder. Therefore, the B7-sample with 100 PPI represents the optimal parameter in this study.

### 3.3. Transducer Preparation and Characterization

To verify the application potential of designed backing materials of Nickel-foam skeleton interpenetrating tungsten-epoxy composite in medical imaging transducers, this study fabricated three 5 MHz ultrasonic transducers: two with a traditional backing and another with the newly designed backing, for comparative analysis. The control group (Transducer B1) utilized a traditional tungsten-powder/epoxy-resin backing (acoustic impedance: 12.10 MRayl, attenuation coefficient: 20.3 dB/cm), while the experimental group (Transducer B7) incorporated the optimal nickel-foam/tungsten-powder/epoxy-resin hybrid composite backing (acoustic impedance: 10.81 MRayl, attenuation coefficient: 62.6 dB/cm). The piezoelectric layer (P5H ceramics, 12 mm diameter) and matching layers were identical for both groups, and the backing layers had a uniform square cross-section of 13.5 × 13.5 mm.

According to the design specifications for medical ultrasonic transducers, a backing layer must provide a total two-way pulse-echo attenuation of at least −60 dB to eliminate trailing artifacts. The theoretical safety thickness (t) required to meet this noise reduction criterion can be derived using the following equation [18]:

(3) $$\mathrm{Total}\;\mathrm{attenuation}\;=\;2\times t\times \alpha \geq 60\;\mathrm{dB}\;\to \;t\geq \frac{300}{\alpha }$$

where t represents the backing thickness (mm) and α denotes the acoustic attenuation coefficient (dB/cm).

**Figure 6:**
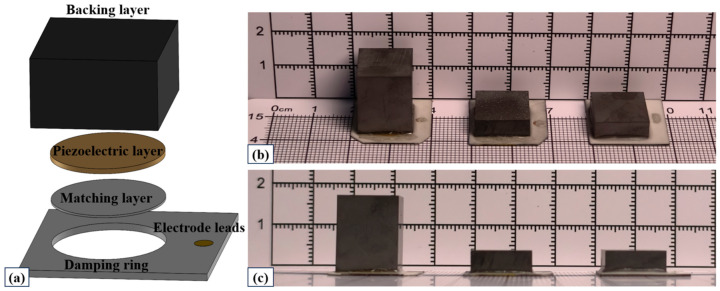
Schematic diagrams and photographs of the transducers. From left to right: B1, B7, and B1-2 models. (**a**) Schematic of overall structure; (**b**) top view; (**c**) front view.

Substituting the measured attenuation coefficients of the different backing materials into Equation (3), the theoretical safety thicknesses are calculated to be *t*_B1_ = 14.77 mm for the control group and *t*_B7_ = 4.79 mm for the experimental group. Considering engineering tolerances and assembly requirements, the fabrication thickness of Transducer-B1 was set to 16.5 mm. In contrast, benefiting from the high attenuation characteristics of the material, the backing thickness of Transducer-B7 was significantly reduced to 5.0 mm. Additionally, for comparative analysis, a control transducer (B1-2) with a traditional backing thickness of 5.0 mm was also fabricated. Photographs of the fabricated transducers are presented in Figure 6.

**Figure 7:**
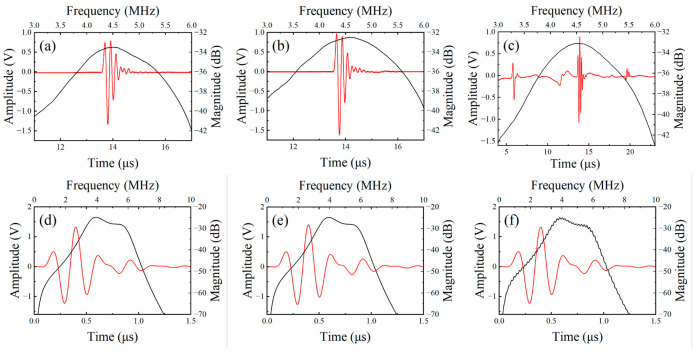
Measured and simulated pulse-echo responses and frequency spectra of the fabricated transducers: (**a**–**c**) measured results for B1 (16.5 mm), B7 (5.0 mm), and B1-2 (5.0 mm); (**d**–**f**) corresponding simulated results. The frequency spectrum in (**c**) is derived from the gated central peak.

The measured pulse-echo responses and corresponding frequency spectra of the fabricated transducers are presented in Figure 7a–c. Specifically, the spectrum in Figure 7c was derived from a Fast Fourier Transform (FFT) of the gated central peak in the time-domain signal. Furthermore, the pulse-echo characteristics and spectra simulated via PiezoCAD 4.0 (Sonic Concepts, Inc., Woodinville, WA, USA) software are shown in Figure 7d–f. The detailed physical and electrical parameters for each transducer are summarized in Table 4. The simulated center frequencies and fractional bandwidths exhibit good agreement with the experimental measurements. The simulated B1 and B7 transducers show comparable performance with minor variations attributed to the differences in backing acoustic impedance, whereas the B1-2 spectrum displays noticeable ripples due to the insufficient backing thickness and damping.

As illustrated in Figure 7, Transducer B7, incorporating the nickel-foam/tungsten-powder/epoxy-resin hybrid composite, effectively suppresses backside interference despite a reduced backing thickness of only 5.0 mm. Its time-domain waveform is notably clear and comparable to Transducer B1, which employs a conventional 16.5 mm backing. In contrast, Transducer B1-2, equipped with a 5.0 mm traditional backing, exhibits severe signal degradation characterized by numerous spurious peaks and an unstable baseline. Quantitative analysis indicates that the spurious peaks at 5.5, 11.5, and 17.5 μs in Figure 7c originate from backward-radiated acoustic waves that were not fully dissipated within the thin backing. Due to the insufficient acoustic absorption of the traditional material at this dimension, the energy undergoes total internal reflection at the backing-air interface and returns to the piezoelectric layer. The 6.0 μs interval between these peaks corresponds precisely to the round-trip travel time within the 5.0 mm backing. Additionally, a weak peak at approximately 20 μs is identified as the secondary pulse-echo reflection between the transducer and the target in the water tank.

Regarding frequency characteristics, the measured center frequencies for all three transducers range from 4.46 to 4.52 MHz, showing minimal variation regardless of the backing material used. However, significant differences are observed in the fractional bandwidth. Transducer B1-2 exhibits the lowest bandwidth at 48.75%, primarily because the 5.0 mm traditional backing (20.3 dB/cm) cannot fully dissipate backward-radiated energy. The unabsorbed waves interfere coherently with the main signal, leading to an uneven spectral energy distribution and a rapid energy drop-off. Conversely, Transducer B7 achieves the widest bandwidth of 63.82%, outperforming Transducer B1 despite being more than three times thinner. This superior performance is attributed to the high attenuation coefficient of the 100 PPI nickel-foam composite (62.6 dB/cm at 5 MHz). While both B1 and B7 eliminate echo artifacts, B7 possesses a significantly higher unit-length attenuation rate. This intense absorption enables faster energy dissipation at the backing interface, effectively shortening the ringing time and broadening the frequency response.

**Figure 8:**
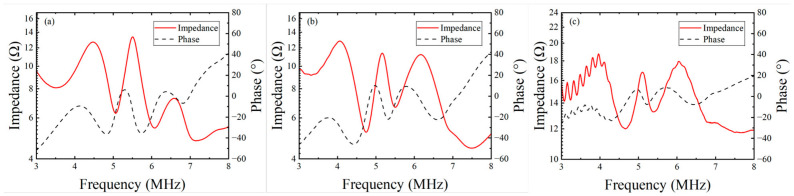
Measured electrical impedance and phase spectra of the fabricated transducers: (**a**) B1 (16.5 mm); (**b**) B7 (5 mm); (**c**) B1-2 (5 mm).

The electrical impedance spectra in Figure 8 further elucidate these physical mechanisms. The resonance peaks around 4 MHz and 5 MHz correspond to the matching layer and the thickness vibration mode, respectively, while the fluctuations near 5.5 MHz originate from the transverse parasitic modes of the 1–3 composite. Transducer B1-2 exhibits a significantly higher impedance magnitude, indicating that mechanical energy is confined within the piezoelectric layer due to insufficient backing attenuation. Its impedance curve shows violent parasitic ripples caused by standing wave patterns from backing-air interface reflections. In contrast, the B7 curve displays a distinct “flattening” feature, as its ultra-high attenuation (62.6 dB/cm) strongly suppresses resonance intensity. While B1 also reduces interference through its 16.5 mm thickness, its resonance peaks remain sharper than those of B7 due to a lower intrinsic attenuation rate. Furthermore, the resonance points of B1 shift toward lower frequencies, reflecting the equivalent mass loading effect exerted by the thick backing.

As summarized in Table 4, Transducer B1-2 yields the highest *k_eff_* (0.447), which is attributed to the minimal mechanical clamping imposed by the weakly damped 5.0 mm backing. In this “free vibration” state, the increased frequency gap between resonance and anti-resonance points results in an artificially elevated *k_eff_*. For high-performance designs, the *k_eff_* of B7 (0.429) is notably superior to that of B1 (0.391). Despite the strong damping characteristics of B7, its electromechanical conversion is enhanced by the superior electrical boundary conditions and conductivity provided by the nickel foam skeleton. Ultimately, B7 achieves a competitive fractional bandwidth of 63.82%, slightly exceeding that of the conventional thick-backing B1.

In summary, the B7 composite enables a 60% reduction in backing thickness, from 16.5 mm to 5.0 mm. Unlike the insufficient B1-2, B7 maintains a clear pulse-echo response and a 63.82% bandwidth, slightly exceeding the thick-backed B1. This demonstrates that the developed material effectively balances high attenuation with compact design for high-performance miniaturized transducers.

## 4. Conclusions

This study designs a novel nickel-foam/tungsten-powder/epoxy-resin hybrid composite backing material that combines high acoustic attenuation with appropriate acoustic impedance. The design utilizes a three-dimensionally interconnected nickel foam skeleton filled with a mixture of tungsten powder and epoxy resin. The acoustic properties of the designed hybrid composite were systematically characterized. Experimental results indicate that the hybrid composite, fabricated using a 100 PPI nickel foam skeleton, achieved acoustic attenuation coefficients of 62.6 dB/cm and 84.2 dB/cm at 5 MHz and 7.5 MHz, respectively. These values are approximately three times higher than those of traditional tungsten-powder/epoxy-resin backing materials. Additionally, the material exhibits an acoustic impedance of 10.81 MRayl, satisfying the impedance matching requirements for medical ultrasonic probes. Furthermore, the practical applicability of the designed hybrid composite was evaluated. In a verification using a 5 MHz medical transducer pair, the transducer equipped with only 5.0 mm of the new backing material achieved a −60 dB two-way pulse-echo insertion loss, effectively eliminating backside interference while maintaining bandwidth performance. Compared to traditional backing materials, the designed backing reduces the axial geometric dimension by over 60%. These results suggest that the backing material designed in this study holds significant potential for application in the future design of miniaturized transducers.

## Figures and Tables

**Table 1:** Physical properties and specifications of the components for the composite backing material.

Material	Parameter	Value/Specification
Nickel foam (Kunshan Xingzhenghong Electronic Materials Co., Ltd., Kunshan, China)	Pores per inch, PPI	20, 30, 40, 60, 80, 100, 130 PPI
	Relative density, *ρ*_r_	<5%
Tungsten powder (Shanghai Macklin Biochemical Co., Ltd., Shanghai, China)	Nominal particle size	3–5 μm
Epoxy (Shanghai Macklin Biochemical Co., Ltd., Shanghai, China)	Resin model	E51
	Curing agent model	W93 Amine-based
	Mass ratio	4:1

**Table 2:** Physical and acoustic properties of Group A samples.

Sample	Pores per Inch, PPI	Relative Density, *ρ*_r_	Average Pore Size μm	Average Strut Diameter μm	Macroscopic Density, *ρ* (g/cm^3^)	Sound Velocity, *c* (m/s)
A1	N/A	N/A	N/A	N/A	1.186	2683
A2	20	3.09%	1270	214	1.414	2675
A3	30	3.04%	847	141	1.405	2668
A4	40	2.86%	635	103	1.407	2668
A5	60	2.74%	423	67	1.385	2641
A6	80	1.73%	318	40	1.317	2620
A7	100	3.28%	254	44	1.436	2633
A8	130	4.57%	195	40	1.527	2535

**Table 3:** Physical and acoustic properties of Group B samples.

Sample	Pores per Inch, PPI	Relative Density, *ρ*_r_	Macroscopic Density, *ρ* (g/cm^3^)	Sound Velocity, *c* (m/s)
B1	N/A	N/A	6.868	1761
B2	20	3.09%	6.612	1749
B3	30	3.04%	6.600	1735
B4	40	2.86%	6.471	1739
B5	60	2.74%	6.275	1745
B6	80	1.73%	6.468	1744
B7	100	3.28%	6.184	1748
B8	130	4.57%	5.400	1765

**Table 4:** Performance specifications of the transducers.

Sample	Measured Center Frequency, *f*_c_ (MHz)	−6 dB Bandwidth, *BW*	Effective Electromechanical Coupling Coefficient, *k_eff_*
B1 (16.5 mm)	4.52	56.96%	0.391
B7 (5.0 mm)	4.46	63.82%	0.429
B1-2 (5.0 mm)	4.50	48.75%	0.447
B1-Simulated	4.53	60.32%	
B7-Simulated	4.55	60.17%	
B1-2-Simulated	4.50	59.33%	

## Data Availability

The original contributions presented in this study are included in the article. Further inquiries can be directed to the corresponding authors.

## References

[1] Szabo T.L. (2013). Diagnostic Ultrasound Imaging: Inside Out.

[2] Hoskins P.R., Martin K., Thrush A. (2019). Diagnostic Ultrasound: Physics and Equipment.

[3] Zhou Q., Lam K.H., Zheng H., Qiu W., Shung K.K. (2014). Piezoelectric single crystals for ultrasonic transducers in biomedical applications. Prog. Mater. Sci..

[4] Rathod V.T. (2020). A Review of Acoustic Impedance Matching Techniques for Piezoelectric Sensors and Transducers. Sensors.

[5] Peng C., Wu H., Kim S., Dai X., Jiang X. (2021). Recent Advances in Transducers for Intravascular Ultrasound (IVUS) Imaging. Sensors.

[6] He Y., Liu X., Zhang J., Peng C. (2023). A Backing-Layer-Shared Miniature Dual-Frequency Ultrasound Probe for Intravascular Ultrasound Imaging: In Vitro and Ex Vivo Validations. Biosensors.

[7] Chen X., Ou B., Liu G., Dai Y., Li B., Dai Y. (2025). Simultaneous enhancement of piezoelectric performance and Curie temperature in high-temperature Bi_4_Ti_3_O_12_ piezoceramics through A/B site co-doping. J. Adv. Ceram..

[8] Li K., Cong S., Bian L., Zhao Z., Wu J., Zhao J., Zhang D., Mei H., Sun E., Qi X. (2024). Simultaneous enhancement of piezoelectricity and temperature stability in Pb(Ni_1/3_Nb_2/3_)O_3_–PbZrO_3_–PbTiO_3_ ceramics via Sm-modification. J. Adv. Ceram..

[9] O’Malley C.J., Tang X., Koval V., Chen K., Wu Z., Banerjee K., Hu W., Yan H. (2025). Unveiling the mechanism of substitution-induced high piezoelectric performance in PLZT ceramics. J. Adv. Ceram..

[10] Grewe M.G., Gururaja T.R., Shrout T.R., Newnham R.E. (1990). Acoustic properties of particle/polymer composites for ultrasonic transducer backing applications. IEEE Trans. Ultrason. Ferroelectr. Freq. Control.

[11] Berg S., Rønnekleiv A. (2012). Acoustic Backing in 3-D Integration of CMUT with Front-End Electronics. IEEE Trans. Ultrason. Ferroelectr. Freq. Control.

[12] Bakarič T., Rojac T., Abellard A.-P., Malič B., Levassort F., Kuscer D. (2016). Effect of Pore Size and Porosity on Piezoelectric and Acoustic Properties of Pb (Zr_0.53_Ti_0.47_) O_3_ Ceramics. Adv. Appl. Ceram..

[13] Tran T.N., Lethiecq M., Levassort F., Pourcelot L. (1996). Experimental Verification of the Theory of Elastic Properties Using Scattering Approximations in (0–3) Connectivity Composite Materials. IEEE Trans. Ultrason. Ferroelectr. Freq. Control.

[14] Hidayat D., Syafei N.S., Wibawa B.M., Taufik M., Bahtiar A., Risdiana (2020). Metal-Polymer Composite as an Acoustic Attenuating Material for Ultrasonic Transducers. Key Engineering Materials.

[15] Qiu Y., Liu J., Lu Y., Zhang R., Cao W., Hu P. (2016). Hierarchical Assembly of Tungsten Spheres and Epoxy Composites in Three-Dimensional Graphene Foam and Its Enhanced Acoustic Performance as a Backing Material. ACS Appl. Mater. Interfaces.

[16] Ma J., Huo D., Qi X., Zhu K., Shen B., Liu Y., Zhang R. (2022). Enhanced electromechanical properties in Pb(Mg_1/3_Nb_2/3_)O_3_–PbTiO_3_ based 1–3 piezoelectric composites using the alternating current poling method. Mater. Sci. Eng. B.

[17] Zhu K., Ma J., Qi X., Shen B., Liu Y., Sun E., Zhang R. (2022). Broadening the bandwidth of ultrasonic transducers via an acoustic impedance gradient matching layer. Sensors.

[18] Goodsitt M.M., Carson P.L., Witt S., Hykes D.L., Kofler J.M. (1998). Real-Time B-Mode Ultrasound Quality Control Test Procedures. Med. Phys..

[19] Cops M.J., McDaniel J.G., Magliula E.A., Bamford D.J., Bliefnick J. (2020). Measurement and Analysis of Sound Absorption by a Composite Foam. Appl. Acoust..

[20] Nguyen H.-Q., Wu Q., Chen H., Chen J.-J., Yu Y.-K., Tracy S., Huang G.L. (2021). A Fano-based acoustic metamaterial for ultra-broadband sound barriers. Proc. R. Soc. A.

[21] Weisser T., Groby J.-P., Dazel O., Gaultier F., Deckers E., Futatsugi S., Monteiro L. (2016). Acoustic behavior of a rigidly backed poroelastic layer with periodic resonant inclusions by a multiple scattering approach. J. Acoust. Soc. Am..

[22] Lu T.J., Hess A., Ashby M.F. (1999). Sound absorption in metallic foams. J. Appl. Phys..

[23] Li Y., Li Z., Han F. (2014). Air Flow Resistance and Sound Absorption Behavior of Open-Celled Aluminum Foams with Spherical Cells. Procedia Mater. Sci..

[24] Wilson D.K. (1993). Relaxation-matched Modeling of Propagation through Porous Media, Including Fractal Pore Structure. J. Acoust. Soc. Am..

[25] Rott N. (1980). Thermoacoustics. Adv. Appl. Mech..

[26] Saleem F., Li S., Cui S., Yao Z., Liu X., Xu T., Bian Y., Zhang Y., Wang S., Yao X. (2025). Microstructural influence on compressive behavior and strain rate sensitivity of open-cell nickel foam. Eur. J. Mech. A Solids.

[27] Treiber M., Kim J.Y., Jacobs L.J. (2009). Correction for partial reflection in ultrasonic attenuation measurements using contact transducers. J. Acoust. Soc. Am..

[28] Kinsler L.E., Frey A.R., Coppens A.B., Sanders J.V. (1999). Fundamentals of Acoustics.

[29] Papadakis E.P. (1965). Ultrasonic attenuation caused by scattering in polycrystalline metals. J. Acoust. Soc. Am..

[30] Sheng P. (2006). Introduction to Wave Scattering, Localization and Mesoscopic Phenomena.

[31] Xu Y., Zhu K., Sun E., Ma J., Li Y., Zheng H., Zhang R., Yang B., Cao W. (2024). Wide-bandwidth immersion ultrasound transducer made of Mn:PIMNT single crystal/epoxy 1–3 composite. Sens. Actuators A. Phys..

[32] Groby J.-P., Lagarrigue C., Brouard B., Dazel O., Tournat V., Nennig B. (2014). Using simple shape three-dimensional rigid inclusions to enhance porous layer absorption. J. Acoust. Soc. Am..

[33] Romero-García V., Theocharis G., Richoux O., Pagneux V. (2016). Use of complex frequency plane to design broadband and sub-wavelength absorbers. J. Acoust. Soc. Am..

